# Effects of ventilation during warm ischaemia followed by ex-vivo lung perfusion in a sheep model

**DOI:** 10.1016/j.clinsp.2026.101104

**Published:** 2026-10-23

**Authors:** Mayara Munhoz de Assis Ramos, Marina Vidal-dos-Santos, Shuqi Yang, Dominique Koerts, Michiel Hu, Roland Hoffmann, Luiz Felipe Pinho Moreira, Michiel Erasmus, Gertrude J. Nieuwenhuijs-Moeke, Ana Cristina Breithaupt-Faloppa, Henri G.D. Leuvenink

**Affiliations:** a Laboratorio de Cirurgia Cardiovascular e Fisiopatologia da Circulação (LIM-11), Instituto do Coração (InCor), Faculdade de Medicina da Universidade de São Paulo, São Paulo, SP, Brazil; b Department of Surgery, University of Groningen, University Medical Center Groningen, Groningen, The Netherlands; c Department of Anesthesiology, University of Groningen, University Medical Center Groningen, Groningen, The Netherlands; d Department of Thoracic Surgery, University of Groningen, University Medical Center Groningen, Groningen, The Netherlands

**Keywords:** Circulatory death, EVLP, Sheep, Transplantation, Ventilation

## Abstract

**Introduction:**

Uncontrolled Donation after Circulatory Death (uDCD) has been considered an alternative to expand the lung donor pool. A key variable in these procedures is whether ventilation is maintained during the ischaemic interval. The present study evaluated the impact of ventilation during warm ischaemia in ovine DCD lungs, followed by functional assessment through Ex Vivo Lung Perfusion (EVLP).

**Methods:**

Ovine lungs were procured from a local slaughterhouse and flushed in situ. Lungs were then assigned to two groups: Ventilated (V) or Non-Ventilated (NV) during 120-minutes of warm ischaemia (WI), followed by 120-minutes of cold storage. EVLP was conducted for 240-minutes using a low-flow strategy, pressure-controlled ventilation, and a custom-made perfusion solution. Lung biopsies and perfusate samples were collected. Histopathological evaluation was performed; ventilatory and perfusion parameters were monitored.

**Results:**

After 240-min of EVLP, both groups presented an increase in the compliance (Cdyn) (p = 0.0001), pO_2_ (p = 0.0047), pCO_2_ (p = 0.0155), and lactate (p = 0.0001), without differences between the groups. Histopathology analysis indicated that haemorrhage after WI was higher in NV (p < 0.001) than V. Inflammatory cytokines gene expression was increased in V vs. NV at the end-EVLP, but no differences were seen, 24h later.

**Discussion:**

Ventilation did not confer a clear advantage in the functional status of the lungs after EVLP and may even be associated with transient increases in inflammatory and apoptotic responses. These findings suggest that immediate inflation alone may be sufficient to preserve graft quality prior to transplantation, and that preservation strategies should not primarily focus on mechanical ventilation.

## Highlights

• Ventilation during warm ischemia does not improve organ functional quality.
• Ventilation during warm ischemia may influence apoptosis prevention.
• Immediate inflation appears sufficient to maintain graft quality prior to transplantation.

**Practitioner Points**

• This study shows that ventilation during warm ischaemia is not required to preserve lung quality evaluated post-EVLP, pointing that simple inflation at procurement may be sufficient.

• These findings may guide optimized preservation strategies, reassure clinicians, and support broader acceptance and use of DCD lungs.

## Introduction

Donation after circulatory death is a strategy to increase the pool of available grafts for transplantation. This is particularly relevant given that over 50% of transplants from deceased donors, in countries such as the Netherlands, are from circulatory dead Donation (D55)^1^^,^^2^ DCD III donors are widely used for transplantation. In this category, ventilation is maintained for as long as possible, except during the agonal and no-touch period, which occurs during withdrawal from life-sustaining therapies in the absence of mechanical or normal ventilation. However, questions remain about the potential DCD I and II donors, not ventilated through Warm Ischemia (WI), being considered. Their viability can be improved through specific interventions and managing the highly complex logistical constraints and unpredictable timing^3^

However, DCD lungs could undergo long periods of oxygen deprivation during WI, contributing to Ischaemia-Reperfusion Injury (IRI) after the reperfusion during transplantation^4^ Since IRI plays a significant role in primary graft dysfunction, leading to post-transplant complications, it is crucial to explore strategies that mitigate its effects. Improving low-quality organs from DCD donors could significantly decrease complications, increase donations, and reduce waiting list mortality.

After procurement, it is normal clinical practice to maintain the lungs at approximately 50% of total lung capacity through clamping to sustain alveolar recruitment, enhance surfactant production, and ensure a limited oxygen supply during transportation. Lungs are, then, flushed and stored in a double sterile plastic bag containing preservation solution and placed in a cooler on ice, maintaining a protective temperature range of 4–8°C. This Static Cold Storage (SCS) preservation technique is known to keep lung viability for up to 8h^5^^,^^6^

Ventilation during the ischaemic period has shown potential to improve graft function,^7^^,^^8^ however we tested the veracity of the immediate mechanical ventilation of donor lungs during warm ischaemia reducing injury and improving the quality of donor lungs, by evaluated the effects of ventilating lungs at the moment of retrieval in a pre-clinical slaughterhouse sheep model. The primary outcomes in this study were the ventilatory parameters; change in lung Compliance (Cdyn) and in pO_2_ during normothermic EVLP, comparing V and NV group.

## Materials and methods

### Animal model and treatment groups

The donation after circulatory death sheep model was developed at the University Medical Center Groningen (UMCG), in partnership with Kroon Vlees, Groningen, a slaughterhouse (where no animal ethics approval was required), aiming to reduce the death of animals for research and following the ARRIVE guidelines. It was used as previously described^9^ Sheep, 3- to 6-months-old, were terminated following electric stunning, exsanguination through the carotid artery. After skinning, the animals were hung for chest and abdominal opening and lung dissection.

Lungs were included if they inflated evenly with a resuscitation bag at 5–10 cm/H_2_O PEEP. Exclusion criteria were lacerations, marked atelectasis, abnormal discoloration, aspiration leaks, or palpable lesions.

### Study groups

Lungs were randomly allocated to 2 groups: V – Ventilated throughout ischaemia (n = 8); NV – Non-Ventilated throughout ischaemia (n = 8) (Fig. S1 – Supplementary Material).

### Preparation

After retrieval, lungs were inspected, intubated, and ventilated with an Ambu bag (21% O_2_, PEEP 5 cm/H_2_O) to resolve atelectasis and assess inclusion. NV lungs were clamped to maintain inflation; V lungs were ventilated with an Oxylog 3000 (frequency 7, pressure 20 cm/H_2_O, PEEP 5 cm/H_2_O, FiO_2_ 50%). Residual tissue and the heart were dissected to expose the Left Atrium (LA) and Pulmonary Artery (PA), followed by a 2L warm (37°C) flush (1L anterograde, 1L retrograde; Table 1 – Supplementary Material). Lungs were then either kept ≥50% inflated (NV) or Ventilated (V) for 2h warm ischaemia. The procurement and transportation were performed during the warm ischaemia phase (Fig. S1 ‒ Supplementary Material). The organs were stored in a sealed plastic bag with the flush solution and placed in a double-containment system consisting of a lidded hard plastic container placed inside an insulated foam box to maintain a stable temperature. The organ (in a plastic bag with flush) was kept on ice at 4°C for 2h while the LA and PA were cannulated, followed by 4h of EVLP.

### Ex vivo lung perfusion

The Lung Assist Machine (XVIVO, Gothenburg, Sweden) was primed with 2L of homemade acellular buffered albumin solution (Table 2, Fig S2, Supplementary Material). While the lungs remained in SCS for 2h, EVLP set-up and perfusate preparation was done. After 2h of SCS, lungs were connected to EVLP, starting perfusion at 0.05 L/min until LA pressure was < 5 mmHg and PA pressure < 15–20 mmHg. Lungs were progressively rewarmed to 37°C by increasing perfusate temperature; ventilation began at 32°C with FiO_2_ 40%. The ventilator was set to pressure-controlled mode (PEEP 5 cm/H_2_O, inspiratory pressure 15 cm/H_2_O above PEEP, rate 7/min). For evaluation, a 30s recruitment manoeuvre with PEEP 10 cm/H_2_O and FiO_2_ 100% was applied every 60 min. During evaluation, deoxygenation gas (86% N_2_, 6% O_2_, 8% CO_2_) was supplied via the membrane oxygenator. EVLP was maintained for 4h, after which it was stopped, and final samples and tissues were retrieved.

### Data and sample collection

The following data were obtained in each experiment: LA pressure, PA pressure, flow and % of flow max and temperature. Data were obtained from ventilator settings or calculated: PEEP, FiO_2_, breathing frequency, tidal volume, minute volume, peak inspiratory pressure, mean pressure and dynamic compliance. As outlined by Spratt et al. (2017)^10^ Dynamic lung Compliance (C_dyn_) was calculated at predefined time points. Perfusate and tissue were harvested at fixed time points and defined locations. Accordingly, sample size estimation was based on Steinkühler et al., 2024, who published data on pulmonary dynamic compliance and oxygenation during ex vivo lung perfusion.

### Perfusate gas parameters

Perfusate was collected from the left ventricle and from the pulmonary artery to determine pCO_2_ and pO_2_ using a gas analyser (ABL555, Radiometer Medical ApS, Denmark) during the perfusion.

The venous PO_2_-to-FiO_2_ (P/F) ratio, measured following a 5-minute stabilization phase, was used for hourly determination of oxygenation capacity. During this period, the arterial connection was deoxygenated using a deoxygenation gas (3L/min) and the lungs were ventilated with an FiO_2_ of 100%.

### Histopathological analysis

Lung tissue fragments were fixed in 4% neutral buffered formalin, processed, paraffin-embedded, sectioned, and stained with haematoxylin and eosin for histopathological evaluation. High-resolution digital scans of the sections were acquired using a C9600 NanoZoomer (Hamamatsu Photonics, Hamamatsu, Japan) and analyzed with ImageJ software. Conventional morphometric analyses were performed on one section per animal (n = 8 per-group), assessing parameters such as interstitial oedema and haemorrhage, evaluated independently by two observers.

### Lung tissue culture (explant-24h)

Lung explant cultures were prepared as described by Fantozzi et al. (2018)^11^ Following EVLP, 2 mm^2^ fragments of lung tissue were incubated for 24 hours in 12-well plates under humidified conditions with 5% CO_2_ at 37°C in Dulbecco’s Modified Eagle Medium (DMEM; Gibco, Thermo Fisher Scientific). Incubated culture media were collected and stored (-80°C) for subsequent analysis. The lung tissue fragments were dried and weighed for dry-to-wet ratio assessment.

### Real-time PCR for gene expression

Gene expression analyses were conducted following Steinkuhler et al. (2024)^12^ Frozen lung tissues were homogenized in lysis buffer (Qiagen, the Netherlands), and total RNA was isolated using the RNeasy Mini Plus Kit (Qiagen, Venlo, the Netherlands) following the manufacturer’s protocol. RNA quality was verified by gel electrophoresis, and concentrations were measured with a Nanophotometer N60 (Implen, Munich, Germany). Complementary DNA (cDNA) was synthesized using random hexamer primers (Promega, Leiden, the Netherlands) and SuperScript III reverse transcriptase (Invitrogen, Breda, the Netherlands). Quantitative PCR was performed with 10 ng of cDNA per reaction using TaqMan primer/probe sets (Thermo Fisher Scientific, Waltham, MA, USA) on a ViiA7 Real-Time PCR system (Thermo Fisher Scientific). Duplicate Cycle Threshold (CT) values were averaged, and expression of TNF-α (Oa04656867_g1), IL-6 (Oa04656315_m1), and IL-1β (Oa04656323_m1) was normalized to the housekeeping gene HPRT1 (Oa04825272_gH) using the ΔCT method, with relative mRNA levels calculated as 2^-ΔCT^.

### Immunohistochemistry of caspase-3

Paraffin-embedded lung tissue sections (3 μm) were rehydrated and subjected to antigen retrieval in EDTA buffer (pH = 8.0) at 95°C for 15-minutes. Endogenous peroxidase activity was blocked using 0.3% H_2_O_2_. Immunohistochemistry was performed with a primary antibody against cleaved caspase-3 (Asp175; Cell Signaling Technology, USA), followed by a 1:50 goat anti-rabbit secondary antibody (Dako, USA) conjugated to horseradish peroxidase and a 1:50 rabbit anti-goat tertiary antibody (Dako, USA). The signal was developed with 3,3’-diaminobenzidine (DAB; Thermo Fisher Scientific, USA) and sections were counterstained with haematoxylin. Negative controls were prepared by omitting the primary antibody. ImageJ software was used to calculate the air space-to-lung tissue ratio and to quantify positively stained cells. Results were expressed as the number of caspase-3-positive cells per tissue mm^2^.

### Analysis of results

Results were presented as mean ± Standard Error of the Mean (SEM) or, for PCR data, as median with 5^th^–95^th^ percentile range. Statistical analyses were performed using GraphPad Prism v.9. Data distribution was evaluated for normality using Shapiro-Wilk test. Comparisons were made using unpaired *t*-tests or two-way analysis of variance. This test was performed, when necessary, after data transformation. It was followed by multiple comparison correction using the two-stage step-up method of Benjamini, Krieger, and Yekutieli to control the false discovery rate and adjusted p values were reported.

## Results

### Perfusate analysis and functional parameters

After 240-min of EVLP, NV both groups presented an increase in the Compliance (Cdyn) (p = 0.0001), pO_2_ (p = 0.0047), pCO_2_ (p = 0.0155), and lactate (p = 0.0001) compared to the V group. It can be noted that both groups ended the EVLP at the same level of oxygenation, pO_2_ (Fig. 1A). Both groups presented similar pCO_2_ levels (Fig. 1B) and produced similar amounts of lactate (Fig. 1C).

**Fig. 1:**
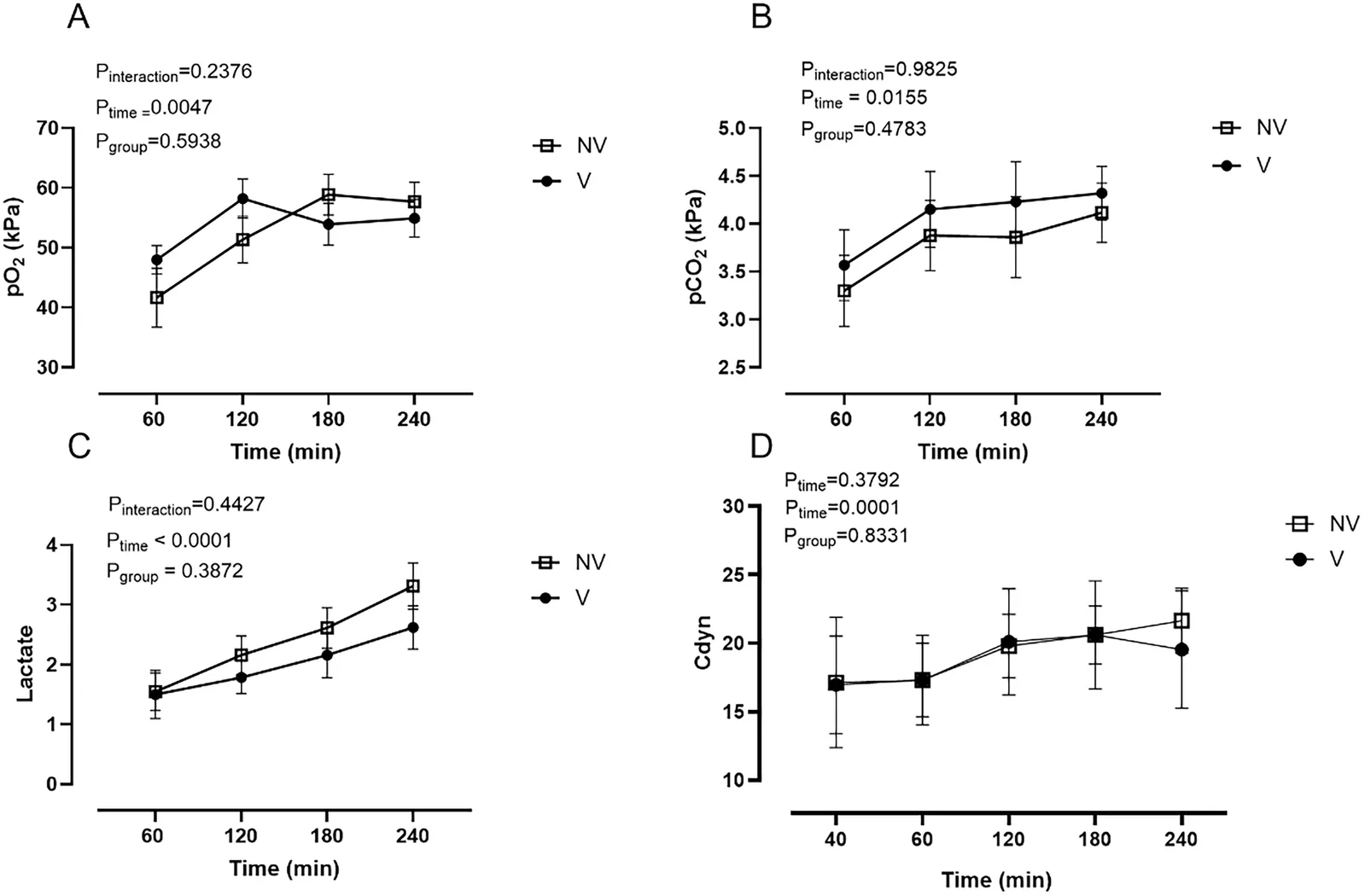
Lung function parameters: (A) PO_2_ p-group = 0.5938, p-time = 0.0047 and p-Interaction = 0.2376, (B) PCO_2_: p-group = 0.4783, p-time = 0.0155 and p-Interaction = 0.9825 (C) Lactate: p-group = 0.3872, p-time < 0.0001 and p-Interaction = 0.3872 (D) Dynamic compliance: p-group = 0.8331, p-time = 0.0001 and p Interaction = 0.3792. The values are presented as the mean ± SEM (n = 8). NV, Non-Ventilated during warm ischaemia; V, Ventilated during warm ischaemia.

Throughout the perfusion time, the compliance results increased Compliance (Cdyn) (p = 0.0001), indicating that lung function (Fig. 1D) increased through the perfusion in both groups in relation to dynamic compliance.

### Histology

After WI, NV presented higher values of red blood cells/mm^2^ (Fig. 2A‒B) when compared to V; however, no significant differences were observed in terms of oedema (Fig. 2C‒D). At the end of EVLP no differences were seen in the oedema, but the haemorrhage remained higher in the NV.

**Fig. 2:**
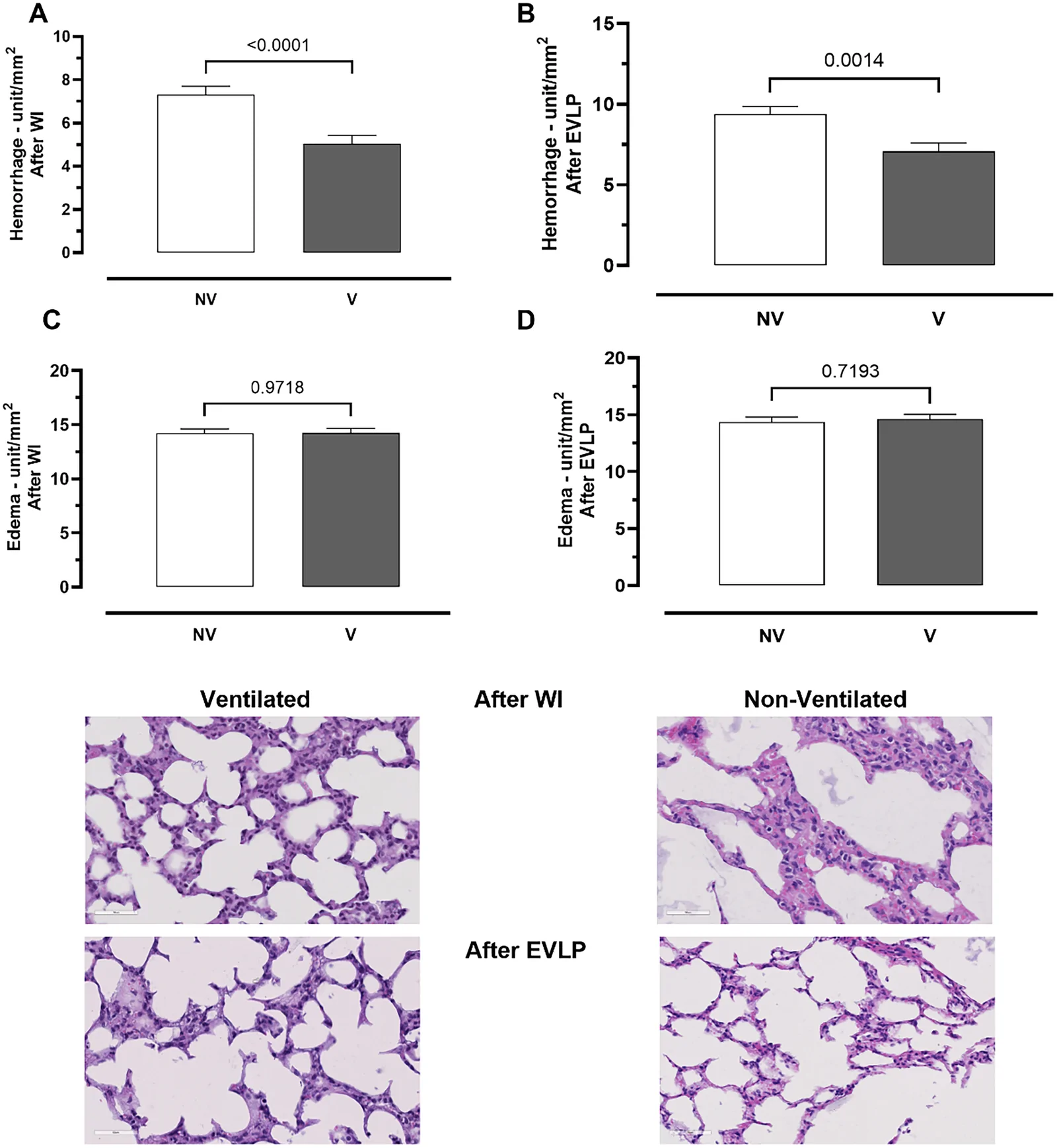
Haemorrhage and oedema in H&E stained biopsies. (A) Haemorrhage after WI p = 0.0223; (B) Haemorrhage After EVLP p = 0.0884; (C) Oedema after WI p = 0.9737; (D) Oedema after EVLP p = 0.8246. The values are presented as the mean ± SEM (n = 8). NV, Non-Ventilated during warm ischaemia; V, Ventilated during warm ischaemia.

### Gene expression in the tissue

TNF-α (Fig. 3A) and IL1-β (Fig. 3B) gene expression did not vary through the timepoints in the NV group. At the end of the EVLP, an increase was observed in the V group. This value was significantly higher than that of the NV group.

**Fig. 3:**
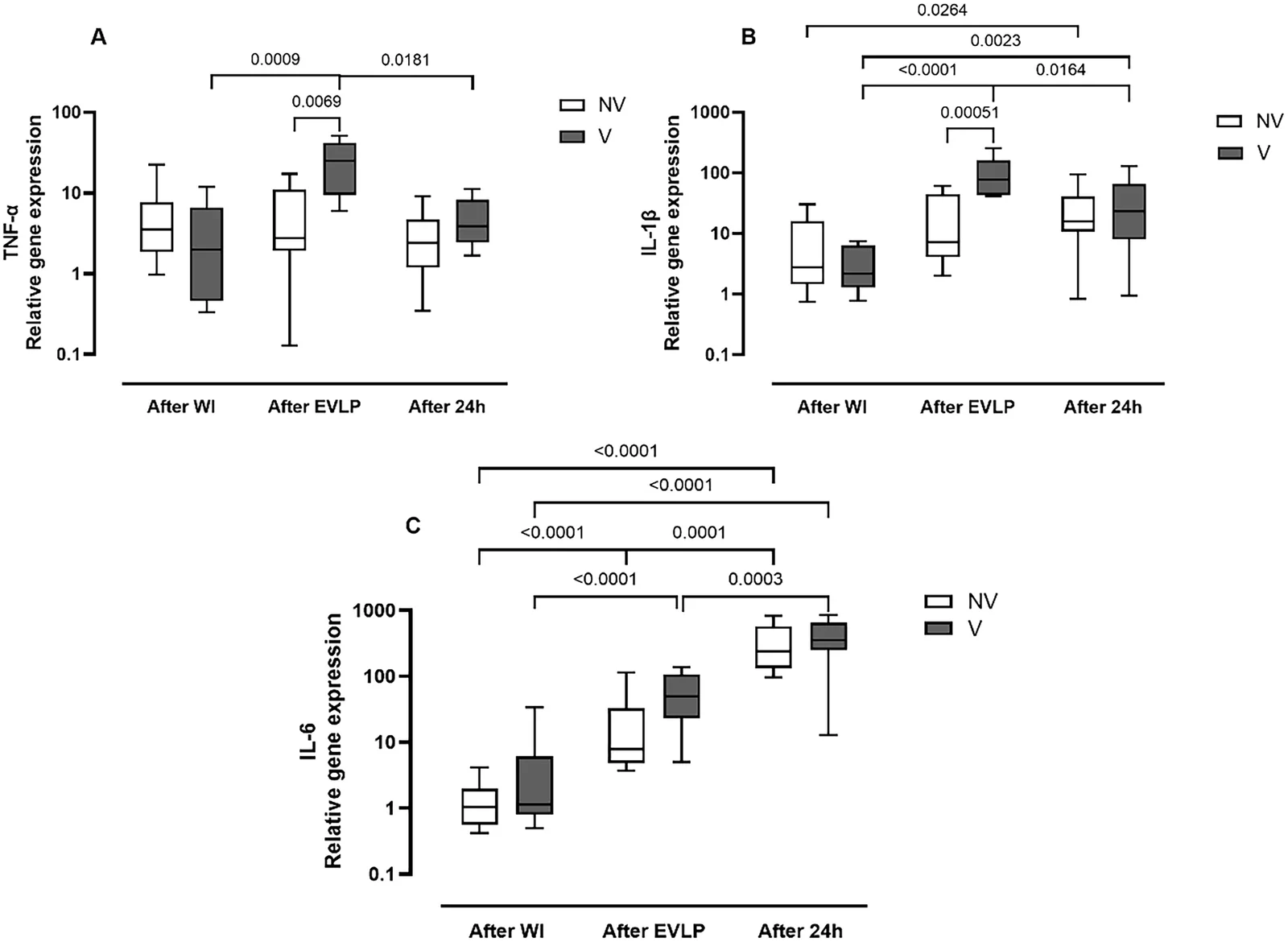
(A) Gene expression of Tumour Necrosis Factor (TNF)-α, (B) Interleukin (IL) 1-β and (C) Interleukin (IL) 6. The values are presented as median and 5%‒95% range (PCR) (n = 8). (A) p-group = 0.0651, p-time = 0.0109, p-Interaction = 0.0114; (B) p-group = 0.1196, p-time < 0.0001, p-Interaction=0.0047; (C) p-group = 0.1063, p-time < 0.0001, p-Interaction = 0.4805. NV, Non-Ventilated during warm ischaemia; V, Ventilated during warm ischaemia.

Despite being continuously elevated, there were no significant differences between the groups. IL-6 did not indicate any differences between the groups, even if there was an expressive increase over the experiment phases.

24h of tissue culture after the end of the perfusion (explant), for TNF-α and IL1-β the gene expression decreased to the same levels seen after WI. Notably, IL-6 expression, in both groups, remained higher than the values found at the end of EVLP (Fig. 3C).

### Apoptosis immunohistochemistry

After the EVLP, the V group presented significantly increased levels of caspase-3-positive cells compared to the NV group. However, after WI and after 24h of tissue culture, both groups presented the same profile (Fig. 4).

**Fig. 4:**
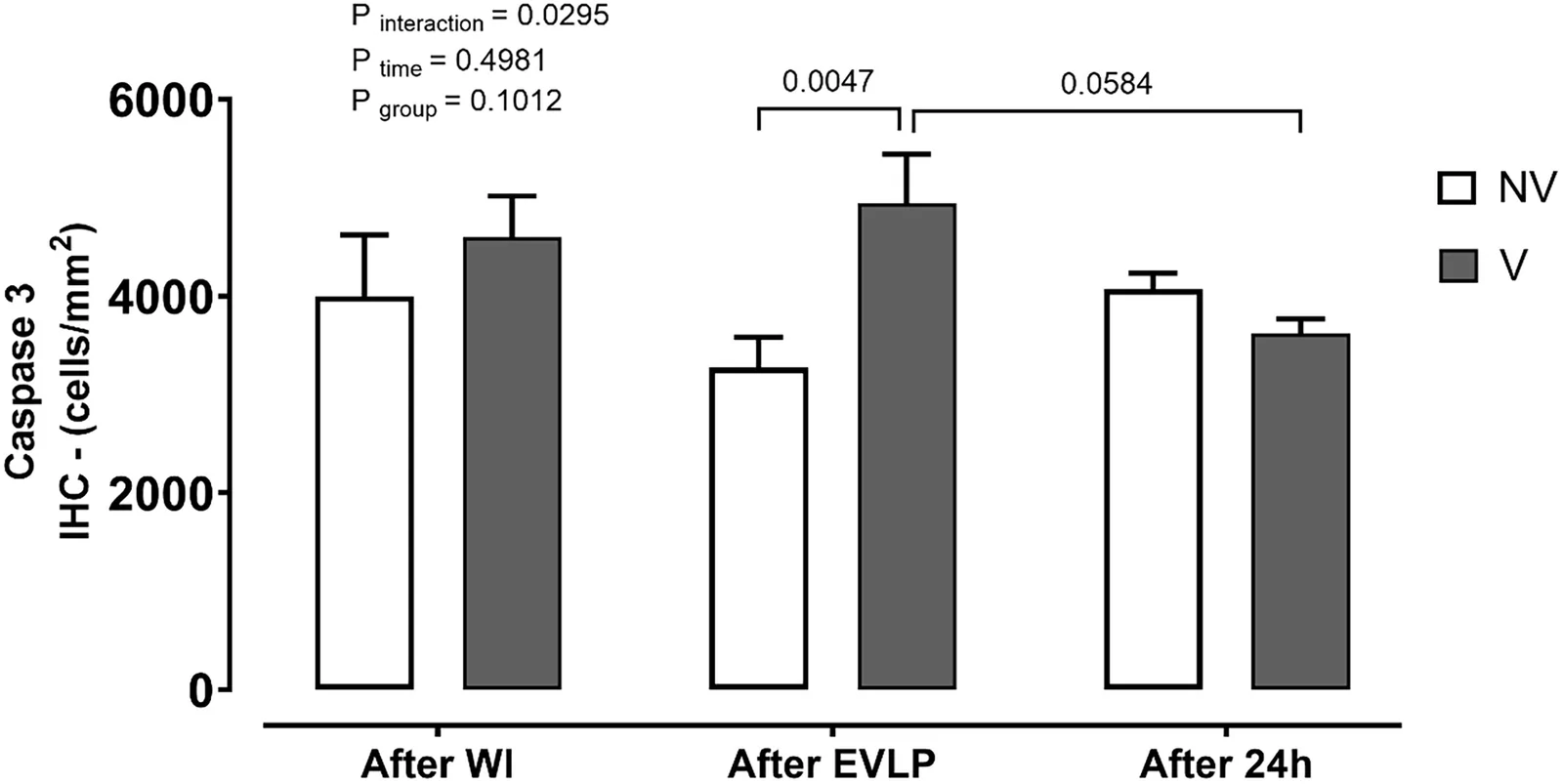
Protein expression of caspase 3 via immunohistochemistry p-time = 0.4981 and p-group = 0.1012 and p-Interaction: 0.0295. The values are presented as the mean ± SEM (n = 6). After a warm flush at the end of EVLP and in the explant tissue culture 24h later. NV, Non-Ventilated during warm ischaemia; V, Ventilated during warm ischaemia.

## Discussion

DCD grafts remain underutilized in clinical practice, making it essential to expand knowledge on this type of donation^13^ Although the number of DCD transplants has doubled over time, they are predominantly liver and kidney transplants^13^^,^^14^ Confidence in DCD lung transplantation remains limited. In a multicentre analysis, EVLP was applied in only 26% of DCD cases, with lungs most often originating from donors who died of anoxia and met extended donor criteria^15^ A major challenge for DCD lungs is the prolonged ischaemia, particularly warm ischaemia, which has been identified as a significant barrier to procurement and management^16^

Additionally, the use of uncontrolled DCD donors has been approached as an alternative to increase the donor pool, and the aspects concerning this donor, such as the longer period of warm ischaemia without ventilation,^2^ are concerning. Clinical experience reports over than 70 lung transplants from uDCD donors Their success depends on variable factors such ischemic times, and preservation techniques. The duration of the WI alters substantially, from 120 to 250 minutes^3^

Experimental evidence suggests that lung inflation during ischaemia may influence metabolic preservation. De Leyn et al. (1993) ^17^ demonstrated that aerobic metabolism could persist for up to four hours in inflated lungs, whereas non-inflated lungs show earlier metabolic decline. However, clinical implementation remains constrained by logistical factors, particularly the requirement for family consent before any intervention. This introduces additional variability in timing. For example, Healey et al. (2020)^18^ reported lung inflation with FiO_2_ of 50% only after consent was obtained, without specifying the delay before intervention.

This study provides insight into the functional and inflammatory profile of DCD lungs after ventilation during WI and during normothermic perfusion. Ventilation during ischaemia was associated with increased apoptosis and elevated expression of inflammatory genes. However, these differences were not sustained after 24 hours, suggesting that early ventilation does not adversely affect longer-term lung quality. Therefore, immediate ventilation under these conditions does not appear to affect long-term lung quality.

Compliance levels align with those observed previously,^9^^,^^12^ without ventilating the lung during WI in a similar slaughterhouse sheep model, demonstrating that ventilation did not influence the compliance. Spratt et al^10^ observed that ventilation during 2 hours of warm ischaemia led to low initial dynamic compliance compared to the group ventilated for 1 hour and lungs procured immediately. On the other hand, the most significant differences observed initially were no longer relevant after 8 hours of machine perfusion. Spratt et al^10^ also pointed out that with 2 hours of donor ischaemia, the effects of ventilation were diminished and occasionally contradictory, with a higher inflammatory profile. Moreover, they suggested that longer periods of WI might lead to injury in the vasculature and tissue, which was aggravated by ventilation after 24 hours of EVLP. They indicate that ischaemia and atelectasis, either individually or in combination, may result in predominantly poor but unpredictable early graft function. Clinically, WI time has not been associated with post-transplant survival, ^19^ and EVLP use is linked to high 1-year survival despite early PGD^19^

After the 4h of EVLP, the V and NV lungs in this study did not show statistically different PaO_2_ values. In contrast, Pierre et al.^7^ and Sakamoto et al.^8^, in pigs and dogs' lungs respectively, showed that the V group tended to present higher PaO_2_ values than the NV group. This lack of alteration in the PaO_2_ in our groups may be due to 2h of ventilation during ischaemia not being enough to cause such effects as seen elsewhere.

Histopathological analysis indicated that NV lungs exhibited more haemorrhage than V lungs after ventilation during WI, consistent with a previous study that successfully transplanted non-ventilated lungs^8^ Oedema in our samples was not affected by EVLP or WI, whereas Sakamoto et al.^8^ reported higher oedema in NV lungs compared to V lungs. Similarly, Pierre et al.^7^ observed greater weight gain in NV lungs after EVLP, though perfusion duration with STEEN™ solution and blood was not clearly reported, potentially accounting for differences. Spratt et al.^10^ also noted greater oedema in NV lungs, and other perfusion models suggest a link between prolonged perfusion and increased oedema. In contrast, our study found no differences in lung weight or oedema before and after perfusion, indicating that ventilation during 2 hours of WI does not directly affect oedema.

Cytokine expression during mechanical ventilation varies based on the ventilation type and severity of lung injury^20^ In Yu et al.,^21^ non-ventilated mice lungs presented higher values of IL-1β gene expression after 24 hours of reperfusion, which contrasts with our results after 4 hours of EVLP, suggesting that longer reperfusion times may be required to better evaluate this parameter. Additionally, contrary to Yu et al.,^21^ our study found higher TNF-α gene expression in the ventilated group after 4 hours of EVLP, possibly due to TNF-α being a more immediate inflammatory response^22^ Our V group exhibited elevated gene expression of inflammatory cytokines at the end of EVLP, which was not observed 24 hours later in tissue culture. Spratt et al.^10^ reported that 2h ventilated groups presented higher perfusate levels of IL-1β and IL-6. Their findings suggest that longer WI times may lead to injury that is exacerbated by ventilation. They concluded that ischaemia and atelectasis lead to lung injury, resulting in unpredictable and poor lung function. Literature has shown that high tidal volume mechanical ventilation does not significantly increase TNF-α levels in Bronchoalveolar Lavage Fluid (BALF) unless there is an additional inflammatory challenge, such as Lipopolysaccharide (LPS) exposure. IL-1β levels are significantly higher in BALF from lungs ventilated with high tidal volumes compared to low tidal volumes or static storage. Ricard, Dreyfuss, and Saumon^23^ observed that high tidal volume ventilation tends to increase IL-1β and IL-6 levels, while TNF-α levels remain low unless there is an additional inflammatory stimulus. Huang et al^24^ indicated that compared to a spontaneous breathing control group, ventilated rats exhibited greater pulmonary permeability, more severe inflammatory cell infiltration and lung oedema, and higher levels of IL-1β, IL-6, and TNF-α in BALF and plasma.

After 2 hours of WI in this study, V lungs presented a higher quantity of caspase-3-marked cells after the EVLP. Forgiarini et al.^25^ suggested that lung ischaemia exceeding 60-minutes may be irreversible due to apoptotic cells becoming necrotic. Furthermore, in humans, the number of apoptotic cells increased with longer reperfusion periods as Fischer et al.^26^ analyzed lung tissue biopsies for apoptosis, showing 21% apoptotic cells, out of the total cells, after 1 hour of reperfusion and 34% after 2 hours. Stammberger et al.^27^ indicated a peak in apoptosis at 2 hours, which then decreased after prolonged reperfusion. Thus, apoptosis might be increased after going through ventilation during WI and is maintained in the long term.

Ventilation did not confer a clear advantage in the functional status of the lungs after EVLP and may even be associated with transient increases in inflammatory and apoptotic responses. These findings suggest that immediate inflation alone may be sufficient to preserve graft quality prior to transplantation, and that preservation strategies should not primarily focus on mechanical ventilation.

## Authors’ contributions

M.M.A.R.: Concept/design; Data collection; Data analysis/interpretation; Drafting article; Funding secured by M. Vidal-dos-Santos S. Yang, H.D. Koerts, M.A. Hu: Data collection, Data analysis/interpretation. R.F. Hoffmann, M. Erasmus, G. J. Nieuwenhuijs-Moeke2, Concept/design, Data analysis/interpretation, Critical revision of article, L.F.P. Moreira, Statistics, Critical revision of article. A.C. Breithaupt-Faloppa, H.G.D. Leuvenink: Concept/design, Critical revision of article, Approval of article.

## Funding

The author M.M.A.R. is a scholarship holder by Coordenação de Aperfeiçoamento de Pessoal de Nível Superior – Brasil (CAPES) ‒ 88887.511368/2020-00 and 88887.716771/2022-00 from Program CAPES-PRINT.

## Data availability statement

The datasets generated and/or analyzed during the current study are available from the corresponding author upon reasonable request.

## Conflicts of interest

The authors declare no conflicts of interest.
