# Supplementary material for: Effects of ventilation during warm ischaemia followed by ex-vivo lung perfusion in a sheep model
Source: Clinics (Sao Paulo). 2026 Oct 23;81:101104. doi: 10.1016/j.clinsp.2026.101104 (PMC13715409; doi:10.1016/j.clinsp.2026.101104)
Supplement: Supplementary file 1 [file mmc1.docx]

**CLINICS-D-26-00600**

**Supplementary Material**

**Table 1** Homemade flush solution.

|  | **1L of solution (mL)** | **mmoL/L** |
| --- | --- | --- |
| **4% succinylated gelatin solution (Gelofusine^®^, B. Braun, Melsungen, Germany)** | 956.51 | 0.00065 |
| **Magnesium sulfate (MgSO4) (100 mg/mL) (Eureco-Pharma B.V., Germany)** | 1.5 | 1.25 |
| **Mannitol (15%) (Baxter B.V., Utrecht, the Netherlands)** | 8.33 | 6.5 |
| **Glucose (20%) (Baxter B.V., Utrecht, the Netherlands)** | 5 | 5 |
| **Calcium Gluconate (10%) (B. Braun, Melsungen, Germany)** | 10 | 2.12 |
| **Potassium Chloride (KCl) (7.45%) (Fresenius Kabi, Germany)** | 5 | 9.89 |
| **Sodium Pyruvate (100 mM, Gibco, Thermo Fisher Scientific)** | 5 | 50 |
| **Trometamol ‒ THAM (0.3 mmoL/mL)** | 0.33 | 0.099 |
| **Nitroglycerine (50 mg/mL) (Hameln pharma gmbh, Hameln, Germany)** | 4 | 0.88 |
| **Heparin (5.000 IE/mL) (LeoPharma A/S, Ballerup, Denmark)** | 2 | ‒ |
| **Vitamin for infusion (Cernevit) in 4.66 mL NaCl (Baxter B.V., Utrecht, the Netherlands)** | 2.33 | ‒ |
| **Sodium phosphate monobasic monohydrate (NaH_2_ PO_4_·H_2_O) (Sigma-Aldrich, Darmstadt, Germany)** | 0.052g |  |
| **Potassium dihydrogen phosphate (KH_2_ PO_4_) (Merck KGaA, Darmstadt, Germany)** | 0.063g |  |

**Table 2** Homemade perfusion solution.

|  | **1L of solution (mL)** | **mmoL/L** |
| --- | --- | --- |
| **Human albumin 20g/100Ml (Prothya Biosolutions B.V.Netherlands, Amsterdam, NL)** | 200 | 0.015 |
| **4% succinylated gelatin solution (Gelofusine^®^, B. Braun, Melsungen, Germany)** | 950.725 | 0.00065 |
| **Magnesium sulfate (MgSO_4_) (100 mg/mL) (Eureco-Pharma B.V., Germany)** | 1.5 | 1.25 |
| **Mannitol (15%) (Baxter B.V., Utrecht, the Netherlands)** | 8.33 | 6.5 |
| **Glucose (20%) (Baxter B.V., Utrecht, the Netherlands)** | 15 | 15 |
| **Calcium Gluconate (10%) (B. Braun, Melsungen, Germany)** | 10 | 2.12 |
| **Potassium chloride (KCl) (7.45%) (Fresenius Kabi, Germany)** | 5 | 9.89 |
| **Sodium Pyruvate (100 mM, Gibco, Thermo Fisher Scientific)** | 5 | 50 |
| **Heparin (5.000 IE/mL) (LeoPharma A/S, Ballerup, Denmark)** | 2 | ‒ |
| **Vitamin for infusion (Cernevit) in 4.66 mL NaCl (Baxter B.V., Utrecht, the Netherlands)** | 2.33 | ‒ |
| **Sodium phosphate monobasic monohydrate (NaH_2_ PO_4_·H_2_O) (Sigma-Aldrich, Darmstadt, Germany)** | 0.052g |  |
| **Potassium dihydrogen phosphate (KH_2_PO_4_) (Merck KGaA, Darmstadt, Germany)** | 0.063g |  |

**Figure S1** Study design. This study included 2 groups (n = 8 each). Sheep DCD lungs underwent 2h of WI under ventilation or no ventilation, followed by Static Cold Storage (SCS) for 2h followed by *ex vivo* lung perfusion, for 4 hours. Lung tissue samples were kept in culture medium for 24h.

**Figure S2** Description of the EVLP system adapted from Steinkuhler, et al., 2024. The Lung Assist (XVIVO^®^, Gothenburg, Sweden) was used as the main equipment in the perfusion. The portable ventilator Oxylog 3000 (Dräger, Germany) was used to ventilate the lungs during the warm ischaemia time enabling it to be ventilated immediately after the procurement; the inflow pressure was measured before reaching the pulmonary artery and when leaving the left atrium.
